# Single-step genomic predictions for crossbred Holstein and Jersey cattle in the United States

**DOI:** 10.3168/jdsc.2023-0399

**Published:** 2023-11-17

**Authors:** A. Cesarani, D. Lourenco, M. Bermann, E.L. Nicolazzi, P.M. VanRaden, I. Misztal

**Affiliations:** ^1^ Department of Animal and Dairy Science, University of Georgia, Athens, GA 30602; ^2^ Dipartimento di Agraria, University of Sassari, Sassari, 07100, Italy; ^3^ Council on Dairy Cattle Breeding, Bowie, MD 20716; ^4^ Animal Genomics and Improvement Laboratory, Agricultural Research Service, USDA, Beltsville, MD 20705

## Abstract

In the dairy cattle sector, the number of crossbred genotypes increased in the last years, and therefore, the need for accurate genomic evaluations for crossbred animals has also increased. Thus, this study aimed to investigate the feasibility of including crossbred genotypes in multibreed, single-step genomic BLUP (ssGBLUP) evaluations. The Council of Dairy Cattle Breeding provided more than 47 million lactation records registered between 2000 and 2021 in purebred Holstein and Jersey and their crosses. A total of 27 million animals were included in the analysis, of which 1.4 million were genotyped. Milk, fat, and protein yields were analyzed in a 3-trait repeatability model using BLUP or ssGBLUP. The 2 models were validated using prediction bias and accuracy computed for genotyped cows with no records in the truncated dataset and at least one lactation in the complete dataset. Bias and accuracy were better in the genomic model than in the pedigree-based one, with accuracies for crossbred cows higher than those of purebreds, except for fat yield in Holstein. Our study shows that genotypes for crossbred animals can be included in a ssGBLUP analysis with their purebred ancestors to estimate genomic estimated breeding values in a single run.

## Highlights

• The number of genotypes of crossbred animals is increasing in US dairy farms.
• Including crossbred data in genomic evaluations is possible.
• This study analyzed purebred and crossbred data together.
• Single-step genomic predictions for crossbred cows were more accurate than predictions based on SNP effects and breed proportions.

## Graphical Abstract

**Figure:**
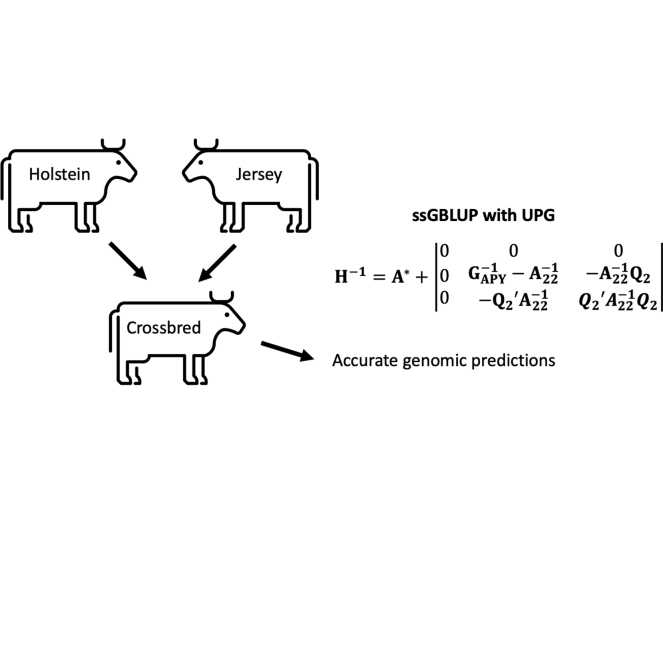
**Summary** The official genetic evaluation for US dairy cattle relies on a multibreed best linear unbiased predictor (BLUP) followed by a single-breed estimation of single nucleotide polymorphism (SNP) effects. The number of genotyped crossbred animals is rising; thus, they should be included in genomic evaluations. This study tested the possibility of including genotypes of crossbred animals in a multibreed single-step genomic BLUP (ssGBLUP) model that considered milk, fat, and protein 305-day yield records from 2000 to 2021. The genomic predictions of crossbred genotyped cows were slightly more accurate than purebred cows.

Multistep evaluations are still the official route to obtaining genomic predictions for dairy cattle in the United States. This evaluation comprises a multibreed BLUP followed by a single-breed estimation of SNP effects (VanRaden et al., 2009). The latter does not include all genotyped individuals, only a reference set. After estimating single-breed SNP effects, direct genomic values (**DGV**) are computed for genotyped animals as a sum of SNP effects weighted by the genotype content. Genomic PTA are then calculated as a linear combination of DGV and parent average (**PA**). This approach has worked well for calculating PTA for purebred animals.

Usually, the routine genomic evaluations for dairy cattle do not consider crossbreeds and are typically made separately by breed. However, there are several studies about genetic and genomic predictions for crossbred cattle (Khansefid et al., 2020; VanRaden et al., 2020; Eiríksson et al., 2021). The main concept behind the predictions of crossbred animals is breed composition (**BC**) or proportion. In the past, a crossbred animal was proportionally assigned to the originating purebreds according to its pedigree. However, the genotyping of crossbred animals strongly increased, and the BC is now based on the genotypes. In the United States, the number of available genotypes of crossbred cattle quickly increased to 150,000 in 2021 and, thus, new concepts were proposed in the genomic era: genomic BC (Hulsegge et al., 2013) and breed base representation (**BBR**; VanRaden and Cooper, 2015). In both methods, the genotype of a crossbred animal is partitioned according to the proportion of the genome originating from each breed and the genomic predictions of the purebreds are usually proportionally combined to evaluate the crossbred animals. In fact, the effects of SNP are usually computed within each purebred and are then applied to the crossbred animals. These concepts were later joined by the concept of breed of origin of the allele (Vandenplas et al., 2016) that can discriminate among the effects of markers due to differences in linkage disequilibrium between markers and QTL (Eiríksson et al., 2021), and it should lead to more accurate predictions than those resulting from the proportional contribution of the purebreds. However, there is no agreement about the advantages of this technique (Xiang et al., 2016; Sevillano et al., 2017).

For crossbreds, VanRaden et al. (2020) recently used BBR, which provides the proportion of DNA contributed by each purebred to a crossbred animal, as a weight for DGV. Although the approach adapts well to the current evaluation system, the reliability of predicting PTA for production traits was only 10% higher than that of PA. A slight gain in reliability for crossbred animals when using SNP effects weighted by BBR indicates that DGV may not result from a simple linear combination of SNP effects from purebred ancestors (Misztal et al., 2022).

Computing SNP effects based on crossbred reference populations in multistep methods could help to increase reliabilities (Steyn et al., 2021); however, this option becomes less straightforward when the breed proportion varies within the population, and there are no clear boundaries between classes to create proper training sets. A different approach to obtaining genomic predictions for crossbred animals is to include their genotypes in the single-step GBLUP (**ssGBLUP**) method (Legarra et al., 2014) that relies on the use of the inverse of a modified relationship matrix (**H**), combining the numerator relationship matrix (**A**) and the genomic relationship matrix (**G**). Therefore, all genotyped and nongenotyped animals can be used together. Steyn et al. (2021) showed that DGV for crossbred animals could be accurately computed when any combination of purebred and crossbred genotypes is included in ssGBLUP. Using data from Holsteins, Jerseys, and their crosses in a single-step approach, Winkelman et al. (2015) obtained genomic EBV with higher reliability for crossbreds than purebreds in some of the traits.

Recently, Cesarani et al. (2022) implemented a multibreed ssGBLUP evaluation for Ayrshire, Brown Swiss, Guernsey, Holstein, and Jersey. The authors analyzed only purebred animals and found that reliabilities from the multibreed model were similar to those from single-breed models. These results were surprising because of the unbalanced number of genotyped animals within each breed: 3.4 million were Holsteins, and only 5k were Ayrshires. In such a scenario, one would expect a decrease in reliability for the least represented breeds because allele effects and frequencies are dominated by the breeds with more data; however, proper modeling of the genetic differences among breeds like nesting the fixed effects within breed helped to avoid loss of predictive power when using only purebred animals. A slight dispersion bias was still present for the least represented breed in the multibreed evaluation, and the authors recommended having one evaluation for Holsteins and Jerseys and another for Ayrshire, Brown Swiss, and Guernsey to avoid bias.

As the number of genotyped crossbred animals in US dairy cattle is rapidly increasing and having accurate genomic predictions for those animals can help make better management decisions in commercial herds, it would make sense to consider them in the evaluation together with their purebred ancestors. Compared with separate evaluations, some studies reported increased reliabilities of this approach in dairy cattle using less than 10k genotyped individuals in ssGBLUP (Winkelman et al., 2015) and less than 50k in GBLUP and BayesR (Khansefid et al., 2020). In this study, we aim to expand on the research findings of Cesarani et al. (2022) and include genotypes for crossbreds in a large-scale, joint Holstein-Jersey ssGBLUP evaluation in the United States.

Data used in the official multibreed genomic evaluations for US dairy cattle breeds were provided by the Council on Dairy Cattle Breeding (Bowie, MD). The data included purebred Holstein (**HO**), Jersey (**JE**), and their crosses (**CROSS**) because these 3 groups are the most sizable (Table 1). Animals were considered purebred if they had more than 90% of their ancestral DNA from the same breed. Crossbred animals were those registered as HO or JE in the pedigree, with one parent of the opposite breed. Owners can report breed codes XD or XX for crossbred dairy animals or report either of the parent breeds. A herd with mostly JE cows may prefer reporting the crossbred progeny as JE, for example, so that the crossbred and purebred EBVs will be reported on the same base as the purebreds in that herd. The analyses considered 305-d milk (**MY**), fat (**FY**), and protein (**PY**) yields for the first 5 lactations recorded from January 1, 2000, to August 2021. All data were preadjusted to have the genetic variance equal across time, breed, and herd and to have the same heritability of 0.20 (Wiggans and VanRaden, 1991; VanRaden, 1997). Data from the last lactation and from lactations not yet completed by August 2021 were projected (VanRaden, 1997).

**Table 1:** Number of records and cows with phenotypes and genotypes^1^

Group	Phenotype		Genotyped animals (only ssGBLUP^1^)
	N	Cows	
Purebred Holstein	42,420,811	18,310,827	1,193,527
Purebred Jersey	4,416,026	1,817,307	224,768
Crossbreds	371,223	155,901	4,215
Total	47,417,185	20,367,132	1,424,863

1 ssGBLUP = single-step genomic BLUP.

Animals were genotyped with 48 different arrays ranging from less than 3k to more than 600k SNPs. Genotypes were imputed, within each breed, to a common set of 79,294 selected SNPs using Findhap v3 (VanRaden, 2015). Crossbreds were imputed separately; genotypes for the purebred parents of all breeds were included to improve imputation. The details on genotype quality control are in Wiggans et al. (2010). Among the 27 million animals in the pedigree, 1.4 million cows with phenotypes and their parents had their genotypes used in the analyses (Table 1).

Two evaluation methods were considered: (1) traditional BLUP and (2) ssGBLUP with unknown parent groups (**UPG**) for **A** and **A**_22_ (Cesarani et al., 2021), where **A**_22_ is the block of the numerator relationship matrix corresponding to the genotyped animals. A total of 16 UPG were considered and defined based on breed (HO or JE), sex, and year of birth (≤2000, 2001–2005, 2006–2010, ≥2011; Cesarani et al., 2022). The algorithm for proven and young (**APY**; Misztal et al., 2014) was used for ssGBLUP with 45,000 animals (according to Cesarani et al., 2022) randomly selected as the core: 36,627 females and 1,035 males for Holstein, 6,920 females and 242 males for Jersey, and 151 females and 25 males for crossbred. For APY, the genotyped animals are split into core and noncore, with recursions based on core animals. This enables a sparse representation of the inverse of the genomic relationship matrix with a much-reduced computing cost. Several studies showed that core animals, which represent the nonredundant genomic information in a population, can be randomly selected (e.g., Fragomeni et al., 2015; Bradford et al., 2017) without reducing the prediction accuracy. The data were analyzed with a 3-trait repeatability animal model that included herd management, age-parity, inbreeding coefficient, and heterosis as fixed effects; UPG as fixed effect; and herd-sire, animal, and permanent environment as random effects (Cesarani et al., 2022). Heterosis was calculated from the full pedigrees going back as many generations as recorded, using the following equation (VanRaden, 1992): 1 − Σ*s_i_d_i_*, where *s_i_* and *d_i_* are fractions of the sire's and dam's genes from breed *i*. For ssGBLUP, all the genotyped animals were used simultaneously in the construction of **G**, which was blended with 5% of **A**_22_ to avoid singularity and include a residual polygenic effect (Hollifield et al., 2022). The mixed model equations were solved by iteration on data and a block preconditioning conjugate gradient using the BLUP90IOD2OMP1 software (Tsuruta et al., 2001).

For the validation, 3 sets were created: (1) purebred HO (n = 688,985), (2) purebred JE (n = 119,743), and (3) CROSS animals (n = 3,235). As in VanRaden et al. (2020), the CROSS group only had cows because most of the crossbred animals are cows that were genotyped to accelerate commercial herd management (e.g., selection of replacement heifers) and the limited number of genotypes available for crossbred bulls. Two datasets were considered: (1) complete, with phenotypes recorded from January 2000 to August 2021, (2) reduced, with phenotypic records up to August 2017. Genotyped cows with phenotypes in the complete but not in the reduced dataset were in the validation set. Three different metrics were used for validation: (1) predictive ability computed as the correlation between phenotypes adjusted for fixed effects (Y_ADJ_) in the complete dataset and the (G)EBV estimated in the reduced dataset; (2) the slope (b1) of the regression of Y_ADJ_ on (G)EBV was used as a measure of the inflation of predictions; and (3) the correlation between (G)EBV estimated in the complete and reduced datasets was used to test the stability of the model when new information is added (Legarra and Reverter, 2018).

Average predictive abilities across traits estimated with BLUP were 0.33, 0.30, and 0.26 for HO, JE, and CROSS groups, respectively (Table 2). As expected, genomic information improved the predictability for all traits and groups. The breeding values estimated in the present paper for purebred HO and JE cows were compared with those estimated in Cesarani et al. (2022) to investigate the impact of including crossbred animals in the analysis. A total of 17.6 million and 1.7 million HO and JE animals were shared between the 2 analyses, and correlations between BV estimated in the 2 studies ranged from 0.98 (MY for JE) to 1.00. The correlation for young bulls was also larger than 0.99. Predictive abilities computed for purebred HO cows in Cesarani et al. (2022), that is, the study without phenotypes and genotypes of crossbred, were 0.55 (MY), 0.55 (FY), and 0.52 (PY). In the present study, the predictive ability for the last 2 traits was 1 point higher. Predictive abilities reported for purebred JE cows by Cesarani et al. (2022) were 0.51 (MY), 0.47 (FY), and 0.52 (PY). The values in the present study were the same, except for FY, which was 1 point lower. As far as the regression coefficients of Y_ADJ_ on EBV from BLUP, the inclusion of crossbred phenotypes led to poorer results compared with Cesarani et al. (2022), whereas values calculated for the 2 purebreds using ssGBLUP were almost the same with or without the crossbred data, suggesting greater stability of the genomic model.

**Table 2:** Predictability, stability, and regression coefficients estimated using BLUP and single-step genomic BLUP (ssGBLUP) for the validation cows

Group	Trait	N	BLUP			ssGBLUP		
			Pred^1^	Stab^2^	b1^3^	Pred^1^	Stab^2^	b1^3^
Purebred Holstein	Milk	688,985	0.30	0.68	0.83	0.55	0.95	1.04
	Fat		0.35	0.74	0.90	0.56	0.96	0.98
	Protein		0.34	0.73	0.88	0.53	0.95	1.01
	Average		0.33	0.72	0.87	0.55	0.95	1.01
Purebred Jersey	Milk	119,743	0.31	0.71	0.91	0.51	0.94	1.08
	Fat		0.26	0.62	0.72	0.46	0.91	0.90
	Protein		0.32	0.70	0.86	0.52	0.93	1.04
	Average		0.30	0.68	0.83	0.50	0.93	1.01
Crossbreds	Milk	3,235	0.37	0.67	0.78	0.66	0.98	1.02
	Fat		0.20	0.44	0.64	0.50	0.92	1.02
	Protein		0.22	0.46	0.75	0.58	0.96	1.09
	Average		0.26	0.52	0.72	0.58	0.96	1.04

1 Predictability = correlation between adjusted phenotype in the complete and breeding values in the reduced dataset.

2 Stability = correlation between breeding values estimated in the complete and reduced datasets.

3 b1 = regression coefficients of adjusted phenotypes on EBV from BLUP and on genomic EBV from ssGBLUP.

The average predictive ability and stability computed using BLUP for crossbred animals were lower than for the 2 purebreds (Table 2). Interestingly, the predictive ability computed for MY in the CROSS group (0.37) was larger than the values for HO (0.30) and JE (0.31). Under ssGBLUP, average values for predictive ability and stability were slightly higher in CROSS (0.58 predictability and 0.98 stability) than in HO (0.55 and 0.95) and JE (0.50 and 0.93) cows. Predictive abilities consider the adjusted phenotypes, which remove the fixed effects from the phenotypes. In the present study, using genomic information within the ssGBLUP model could have partially overcome the absence of breed as a fixed effect. Assuming that accuracies are inflated for crossbreds due to incomplete accounting for BC, the inflation can be reduced by better accounting for this effect (Misztal et al., 2022).

While many studies account for the effect of heterosis, the recombination loss is usually ignored. If the exact type of the crossbreed can be determined (e.g., F_1_ or F_2_), that effect can added to the model. If such a determination is complex (e.g., due to incomplete pedigree information), such a determination can be attempted based on the genomic information. In the end, the inflation may not be detrimental in practice if the genomic EBV of crossbred animals is mainly used for management decisions, and increased predictivity allows for predicting future phenotype of the animals better. The higher accuracies for crossbreds in MY could be explained by the larger phenotypic difference between HO and JE, reflecting a greater genetic difference between the 2 originating breeds. These breed differences, which can be easily predicted from the genotyped animals, can contribute to larger reliabilities in the crossbred population in a scenario where the genomic predictions of crossbred animals are weighted according to each breed's DNA proportion (VanRaden et al., 2020). The higher accuracy reported for crossbred animals is not uncommon in dairy cattle (Winkelman et al., 2015; Khansefid et al., 2020) and other species (Hidalgo et al., 2016).

Winkelman et al. (2015) focused on Holstein, Jersey, and their crosses in New Zealand. In their study, predictions for crosses were consistently more accurate than for Jersey, except for longevity. In the study of Winkelman et al. (2015), the average accuracy of genomic EBV for crosses was lower than those estimated for Holstein for fat, fertility, and longevity but higher for the other 4 traits under investigation. Crossbred dairy cattle had higher accuracy when their data were considered in the reference population (Khansefid et al., 2020). VanRaden et al. (2020) reported the correlations of yield deviation on (G)PTA for 10,124 genotyped crossbred cows: the values were 0.64 (MY), 0.42 (FY), and 0.47 (PY) without genomics, and 0.68 (MY), 0.48 (FY), 0.51 (PY) with genomics. The values found for BLUP in the present study are lower probably because differences in unknown parent grouping or in data truncation; on the contrary, values estimated in the present study using genomics are equal to or larger than those reported by VanRaden et al. (2020), indicating that benefits from directly including the genomic information in a single step exceeded any initial disadvantage in pedigree modeling. In the latter study, the improvement with genomics varied according to the BBR of the crossbred cows: the largest increase was observed for cows with BBR between 75% and 89%. The average improvement using genomics reported by VanRaden et al. (2020) is much lower than the improvements found in the present study. The reason could be linked to the different methods used to compute the genomic EBV—ssGBLUP in the present study and multistep in VanRaden et al. (2020)—since the number of considered genotypes was similar in both studies. This difference in methodology could also be observed in that ssGBLUP better predicted crossbred cows because HO and JE differ greatly in milk yield, causing larger genetic variance among the different crosses.

For dairy cattle, inflation values of 1 ± 0.15 are still acceptable (Tsuruta et al., 2011). According to the Interbull validation, the b1 should range from 0.90 to 1.10 for large populations or be within statistical significance from 1.0 for smaller populations (Interbull CoP, Appendix VIII; Interbull, 2011). The b1 values estimated with ssGBLUP were all within 1 ± 0.1. The average value was 1.02 ± 0.06, ranging from 0.90 to 1.09, whereas for BLUP, the EBVs were more inflated (0.81 ± 0.09) and with a more extensive range (0.72–0.91). In ssGBLUP, all validation groups showed nonbiased average predictions. The average values for each group were 1.01 ± 0.03 for HO, 1.01 ± 0.09 for JE, and 1.04 ± 0.04 for CROSS. In VanRaden et al. (2020), the regression coefficient estimated for genotyped crossbred cows were 0.83 (MY), 0.91 (FY), and 0.84 (PY) for the pedigree model and 0.95 (MY), 1.04 (FY), and 0.97 (PY) using genomics. The number of genotyped animals considered in the present study was very similar to VanRaden et al. (2020) but larger than other studies (Winkelman et al., 2015; Khansefid et al., 2020). Khansefid et al. (2020) analyzed the accuracy and bias of predictions using different reference sets and prediction strategies: inflated genomic predictions were reported for both purebred and crossbred animals. The inflated breeding values estimated in most scenarios could be associated with a nonoptimal reference population and a small number of crossbred genotypes that did not allow a good representation of this group of animals. The importance of the reference population was reported also by van den Berg et al. (2020). Probably, a more precise definition of the crossbred animals (i.e., identification of breed proportions) could lead to more accurate predictions.

Before the genomic era, a reliable and deep pedigree was necessary to assign the proportion of the genotype of a crossbred individual to the originating breeds. Nowadays, allocating each DNA segment to the originating purebred based on genotypes is more accurate. However, accurately identifying a specific breed origin for each SNP can be challenging to implement in routine evaluations. In the present study, genotypes of purebred and crossbred were considered together, and **G** accounted for the relationship among them. If phenotypes for all breeds are on the same scale, then SNP effects can be estimated by considering all the genotypes together. Genomic predictions of less numerous breeds and crossbred animals from ssGBLUP could be worsened in case of an imbalanced number of genotypes among breeds because the more represented breeds could dominate the allele effects and frequencies. In the present study, crossbred animals represented less than 1% of the genotyped animals, and most of them (about 80%) were considered validation animals. Thus, their impact on the evaluation of purebred animals is negligible. On the contrary, including crossbred and purebred data in a ssGBLUP model could enhance the prediction of crossbred animals through the **H** matrix. Moreover, the impact of including a fixed number of purebred and crossbred animals in the core for APY deserves further investigation.

The genomic setup took about 10 h, whereas the EBV computation took around 4 h (408 rounds, 33.32 s each). The solving process for ssGBLUP needed 3 more hours (336 rounds, 69.90 s each). This means that the whole genomic process could be carried out in less than one day for these 3 traits. These computing times (time to reach the convergence, i.e., preconditioned conjugate gradient with iteration on data) are lower than those reported by the recent studies involving similar datasets of US dairy cattle (Cesarani et al., 2021, 2022) due to the implementation of a new method for including a residual polygenic effect in the model (Hollifield et al., 2022). Further computational improvements might be achieved by indirectly predicting young genotyped animals (Tsuruta et al., 2021) or by using solutions from previous runs (Winkelman et al., 2015).

Crossbred data can be included in multibreed US dairy cattle single-step evaluations without reducing the accuracy or increasing the inflation of genomic EBV for purebred animals. This evaluation system allows similar gains in accuracy for purebreds and crossbreds. Additionally, the inclusion of crossbred animals into the single-step run simplifies the genetic evaluation pipelines and increases computing efficiency while delivering predictions for managing commercial crossbred herds.
